# Idiopathic Hypertrophic Pyloric Stenosis with Complete Ladd's Band: A Rare Association

**DOI:** 10.1055/s-0039-1698400

**Published:** 2019-11-22

**Authors:** Ahmed M. Abo Elyazeed, Mohamed M. Shalaby, Mohamed M. Awad, AbdelMotaleb M. Effat, Ahmed E. Abdella, Sherif Mohamed Shehata

**Affiliations:** 1 Division of Surgery, Department of Pediatric Surgery, Tanat University Hospital, Tanta, Gharbia, Egypt

**Keywords:** laparoscopic pyloromyotomy, malrotation, Ladd's band, Ladd's procedure, laparoscopic exploration, postoperatively, vomiting

## Abstract

A male infant aged 45 days presented with projectile nonbilious vomiting for 2 weeks. Ultrasound showed picture of idiopathic hypertrophic pyloric stenosis. Laparoscopic pyloromyotomy was done, but postoperative vomiting that was mainly nonbilious continued without improvement. After 4 days of persistent vomiting, laparoscopic exploration was done and complete pyloromyotomy was confirmed and malrotation with complete Ladd's band was found, then case converted to open laparotomy and Ladd's procedure was done. Postoperatively, vomiting stopped completely and baby began gradual feeding till reaching full feed. Despite that the presentation of concurrent Idiopathic Hypertrophic Pyloric Stenosis with malrotation is extremely rare; a formal laparoscopic abdominal exploration should be done as the first step before proceeding to pyloromyotomy.

## Introduction

Infantile hypertrophic pyloric stenosis (IHPS) is a common problem that is often seen in daily care in the pediatric surgical units. 1 The incidence of IHPS is ∼1 to 3 per 1,000 live births. 2 It is more often in males, 3 with a male-to-female ratio of 4:1. 3 Laparoscopic approach to IHPS was introduced in 1991. The potential advantages of the laparoscopic pyloromyotomy are shorter hospital stay, better cosmoses, shorter postoperative recovery, lower complications rate, and less postoperative pain. 4 5 Ladd's band is a problem affecting neonates with incidence of 1 per 500 live births. 6 Ladd's band with abnormal mesenteric attachments and a narrowed mesenteric base can lead to midgut volvulus. 5 7 Simultaneous presence of pyloric stenosis and malrotation had rarely been found in the literature. 7 8

## Case Report

**Fig. 1:**
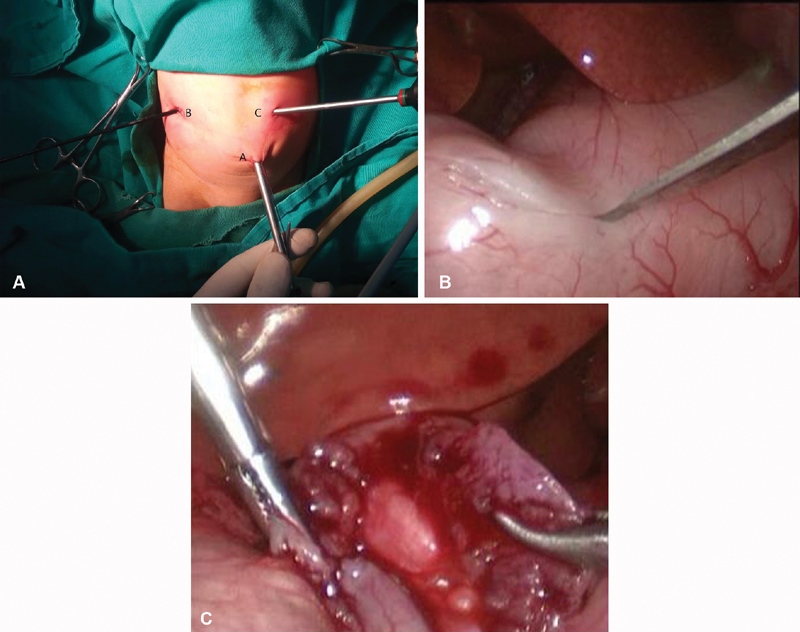
( **A** ) Operative photos showing the ports position in Tan's approach where A is umbilical port (telescope), B is duodenal grasper, and C is knife and spreader. ( **B** ) Operative photo of laparoscopic view showing the myringotomy knife incising the pyloric mass of the pylorus. ( **C** ) Operative photo of laparoscopic view after complete pyloromyotomy with shoe shine maneuver showing the incised thickened muscle with no obstruction with clear mucosa.

**Fig. 2:**
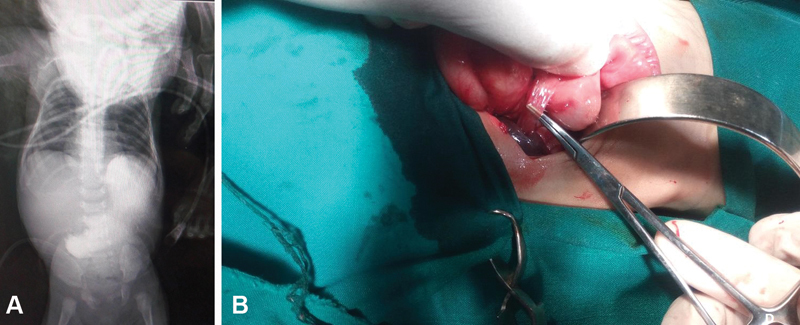
( **A** ) Radiologic photograph of abdomen with oral contrast postoperatively showing the retained contrast in the stomach. ( **B** ) Operative photo in the second surgery shows cleavage of the Ladd's band.

A 45-day-old term male infant was born by normal vaginal delivery. He presented with projectile nonbilious vomiting for duration of 2 weeks. He had a history of poor weight gain and refusal of feeding. On examination, he was dehydrated and abdomen was lax and not distended; ultrasound showed classic picture of pyloric stenosis, the wall thickness was 7 mm, and the length of pyloric canal was 19 mm. Laboratory investigation revealed hypochloremic, hypokalemic metabolic alkalosis; so the patient was admitted for correction of electrolyte disturbance and rehydration. Under prophylactic antibiotics and endotracheal intubation, we placed a 5 mm camera trocar and introduced a 30° scope. Two stab incisions were made in the left and right upper abdomen according to Tan. Right upper quadrant incision of the abdomen for duodenal grasper and left upper quadrant of the abdomen for knife and spreader were performed ( Fig. 1A ). Complete pyloromyotomy was performed ( Fig. 1B and C ). However, in the early postoperative period vomiting persisted. Therefore, 4 days later a contrast study was done that was nonconclusive as it showed delayed passage of the contrast material to small intestine ( Fig. 2A ). We decided to perform relaparoscopy, which confirmed complete pyloromyotomy. However, during this second procedure. complete Ladd's bands without malrotation were found. After conversion to an open procedure due to vital instability, a Ladd's procedure was done ( Fig. 2B ). Postoperatively, all symptoms completely resolved and baby began gradual feeding till reaching full feed and discharged home in the third postoperative day from the Ladd's procedure.

## Discussion

The classical operation for IHPS is Ramstedt pyloromyotomy; however, laparoscopic pyloromyotomy is a minimally invasive version of the Ramstedt procedure that has been associated with a lower incidence of postoperative emesis and a shorter hospital stay, but occasionally results in incomplete pyloromyotomy. 9 10 11

Feeding can be resumed in most infants within a few hours after surgery. Regurgitation occurs in as many as 80% of infants after pyloromyotomy and should not delay feedings. Vigorous postoperative vomiting is infrequent. In a meta-analysis, infants offered feedings 4 hours after operation tolerated full feedings sooner and had a shorter hospital stay compared with infants receiving an incremental feeding schedule, despite having more emesis episodes. 12 13 14 Radiologic evaluation should be performed if vomiting persists beyond 5 days postoperatively, 9 with the understanding that interpretation of the study may be difficult because of postoperative swelling. 11 We did a contrast study to the patient where it showed a delay in the passage of the contrast to the small intestine. This delay is explained by that the vomiting was not projectile in each time denoting incomplete obstruction. So, we decided to proceed for laparoscopic exploration as the contrast study was nonconclusive but showed delay in the passage of the contrast to the small intestine. Persistence of vomiting post pyloromyotomy usually is due to incomplete pyloromyotomy 15 16 but here we show that it may be due to other cause like in this case of associated Ladd's band that is rare but can be found.

The association of concurrent idiopathic hypertrophic pyloric stenosis with malrotation is rare in the pediatric literature. In 1991, Croitoru et al described three cases of malrotation that were associated with pyloric stenosis. 7 The first patient was diagnosed with upper gastrointestinal series obtained because of a high index of suspicion, while the second and third cases were detected by vomiting following Ladd's procedure. 7 In 2008, Bhalla et al described another case that discovered accidentally during preoperative contrast study. 17

To our knowledge, this is the first reported case of IHPS associated with Ladd's band. IHPS may be associated with other anomalies like congenital hernia and malrotation as described in four cases in literature, so we recommend that laparoscopic formal abdominal exploration should be done as the first step before proceeding to pyloromyotomy that can be excluded or managed in the same session using the advantages of the laparoscope saving second general anesthesia exposure.

## Conclusion

Despite that the presentation of concurrent IHPS with malrotation or congenital bands is rare but it exists, so formal laparoscopic abdominal exploration should be done as the first step before proceeding to pyloromyotomy.

## Notes

New Insights and the Importance for the Pediatric Surgeon When performing a laparoscopic pyloromyotomy, laparoscopic abdominal exploration should be the first step of the procedure so any associated lesion can be dealt with in one session.
